# Supplementary material for: The effect of antibiotic selection on collateral effects and evolvability of uropathogenic Escherichia coli
Source: NPJ Antimicrob Resist. 2024 Jul 17;2:19. doi: 10.1038/s44259-024-00037-4 (PMC11254750; doi:10.1038/s44259-024-00037-4)
Supplement: Supplementary file 1 — Supplementary Information [file 44259_2024_37_MOESM1_ESM.pdf]

Supplementary Table S1: Disc diffusion of cephalexin (CL), ciprofloxacin (CIP), gentamicin (CN), meropenem (MEM), nitrofurantoin (F), piperacillin/tazobactam (TZP), trimethoprim (W), cefuroxime (CXM), amoxicillin-clavulanic acid (AMC), tetracycline (TE), ampicillin (AMP), sulphamethoxazole (RL) and Fosfomycin (FOT) to UTI-34, UTI-39 and UTI-59. All results in millimetres and represent a single replicate. Resistance according to EUCAST breakpoints is indicated by red text. A single biological replicate was performed for each antibiotic-isolate combination (*n*=1).

	**CL**	**CIP**	**CN**	**MEM**	**F**	**TZP**	**W**	**CXM**	**AMC**	**TE**	**AMP**	**RL**	**FOT**
**UTI-34**	19	39	28	33	20	20	24	21	17	0	0	9	35
**UTI-39**	22	40	16	36	23	30	25	27	24	25	14	0	37
**UTI-59**	22	36	23	37	28	27	24	24	23	23	14	12	37

Supplementary Table S2. Numbers of publicly available genomes in which the FolA coding mutations obtained in this study were identified. Genomes are then classified based on their host association and sample source.

	**D27E**	**W30R**	**I94L**	**P21L**	**L26R**	**W30G**
**Genomes**	27	273	27	41	9	7
**Human associated**	8	160	10	13	5	5
**Human with source**	4	115	9	7	2	1
**Human urine**	2	26	3	6	2	0

Supplementary Table S3: Accession numbers for each isolate sequenced using the Oxford Nanopore Technologies and/or Illumina sequencing platforms under the BioProject number PRJNA1037559.

Accession number	Sample Name
SAMN38186228	UTI-34
SAMN38186229	UTI-39
SAMN38186230	UTI-59
SAMN38186231	34A
SAMN38186232	34B
SAMN38186233	39A
SAMN38186234	39B
SAMN38186235	39C
SAMN38186236	39D
SAMN38186237	59A
SAMN38186238	59B
SAMN38186239	59C
SAMN38186240	59D

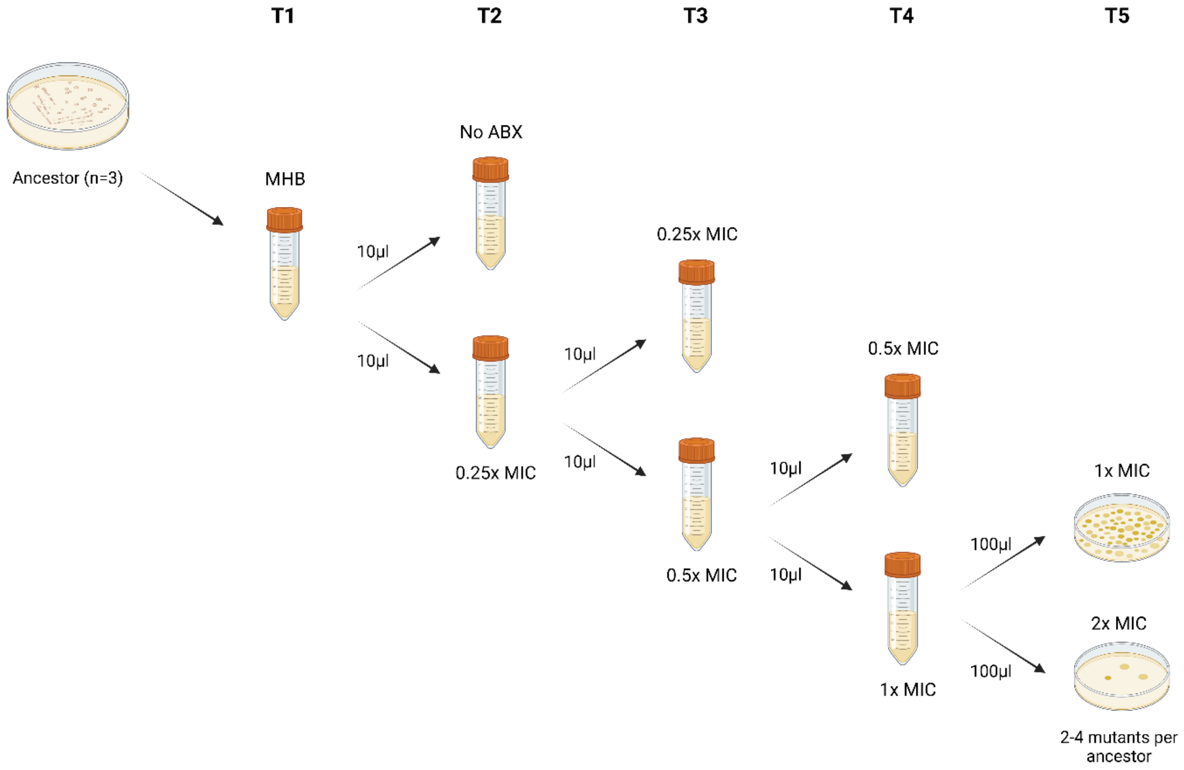

Supplementary Figure S1: Diagram of the evolutionary ramp experiment to select for trimethoprim-resistant derivatives from the three clinical isolates of *Escherichia coli*. Image was created using BioRender.

A

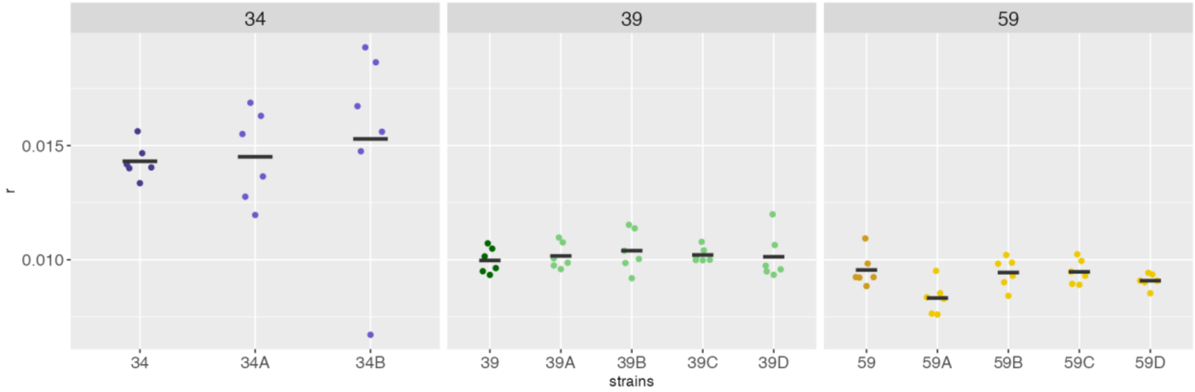

B

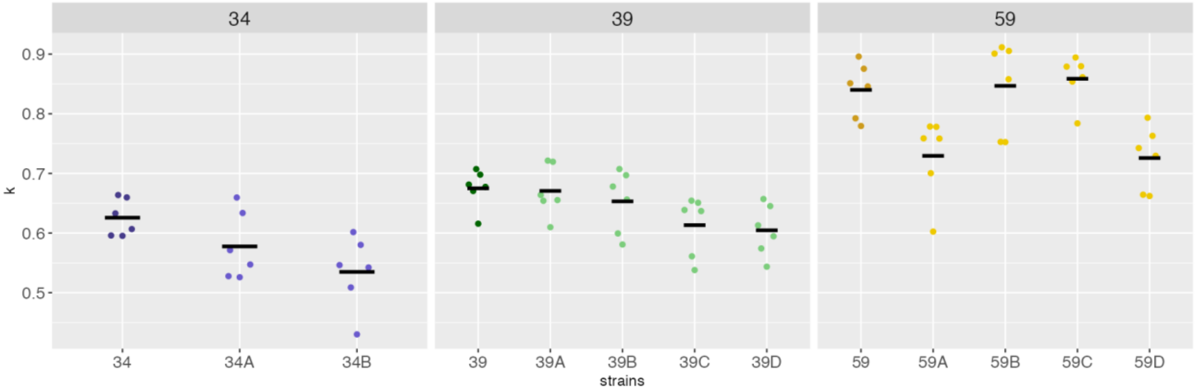

C

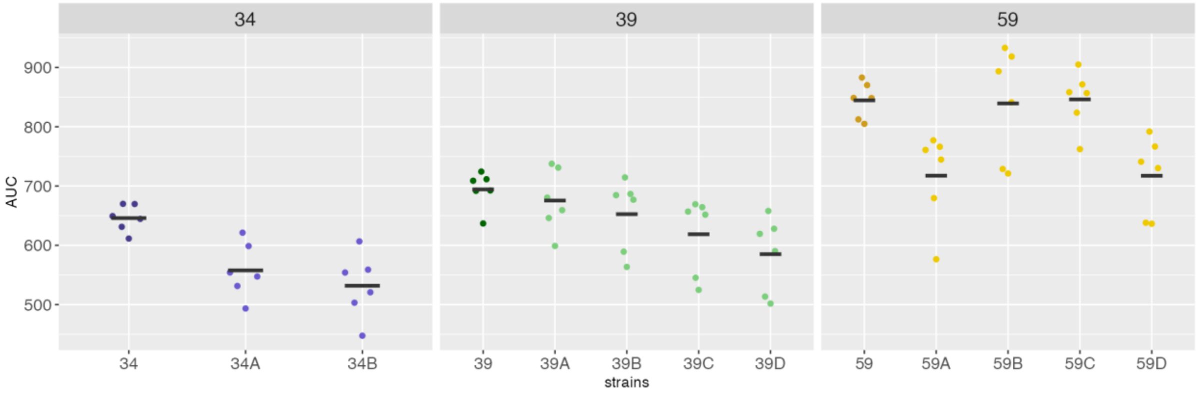

Supplementary Figure S2: Plots of A) maximum growth rate (*r*), B) carrying capacity (*k*) and C) area under the curve (AUC) of the trimethoprim-resistance derivatives relative and the ancestor clinical isolates. Three biological replicates and two technical replicates were performed for each isolate (*n*=3).

A

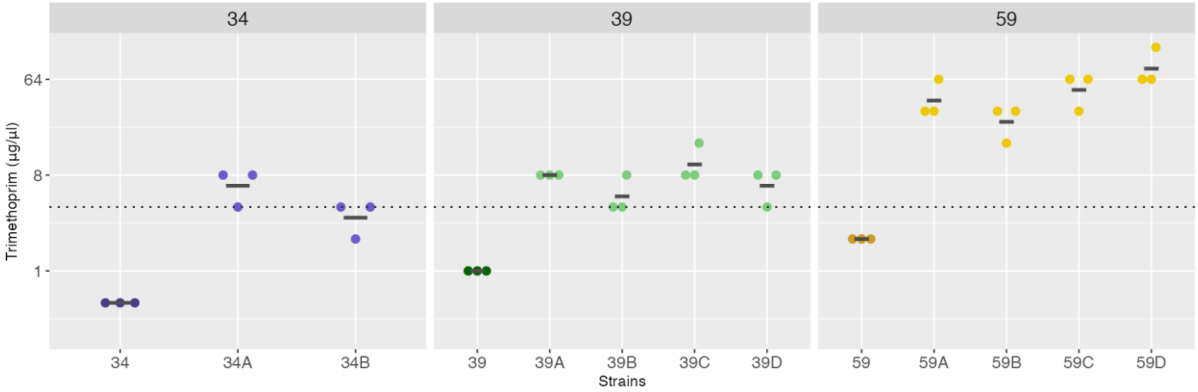

B

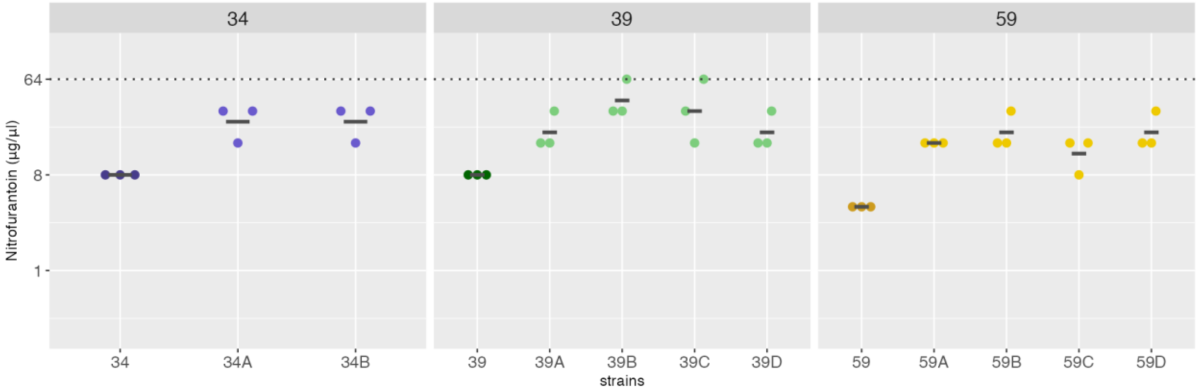

C

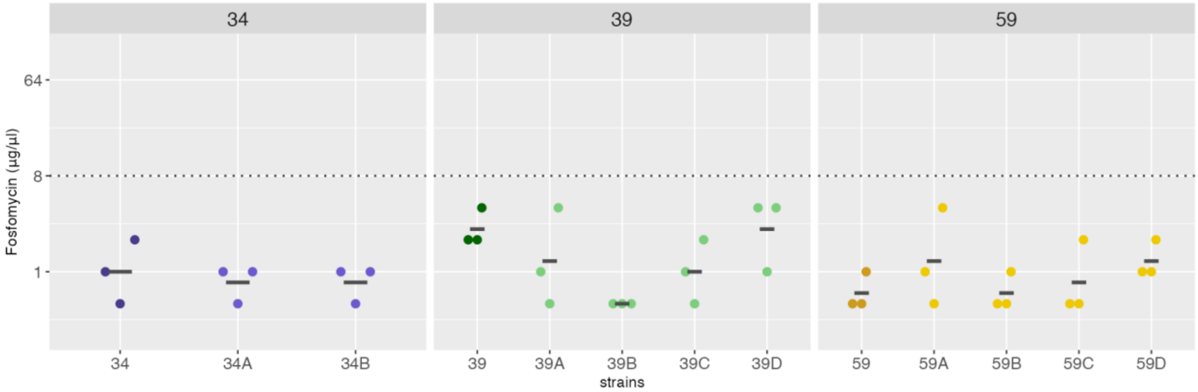

Supplementary Figure S3: Minimum inhibitory concentrations of ancestor clinical isolates and trimethoprim-resistant derivatives for A) trimethoprim, B) nitrofurantoin, and C) fosfomycin. Dotted lines represent the clinical breakpoint for the corresponding antibiotic and line for each group represents the mean. Three biological replicates were performed for each antibiotic-isolate combination (*n*=3).

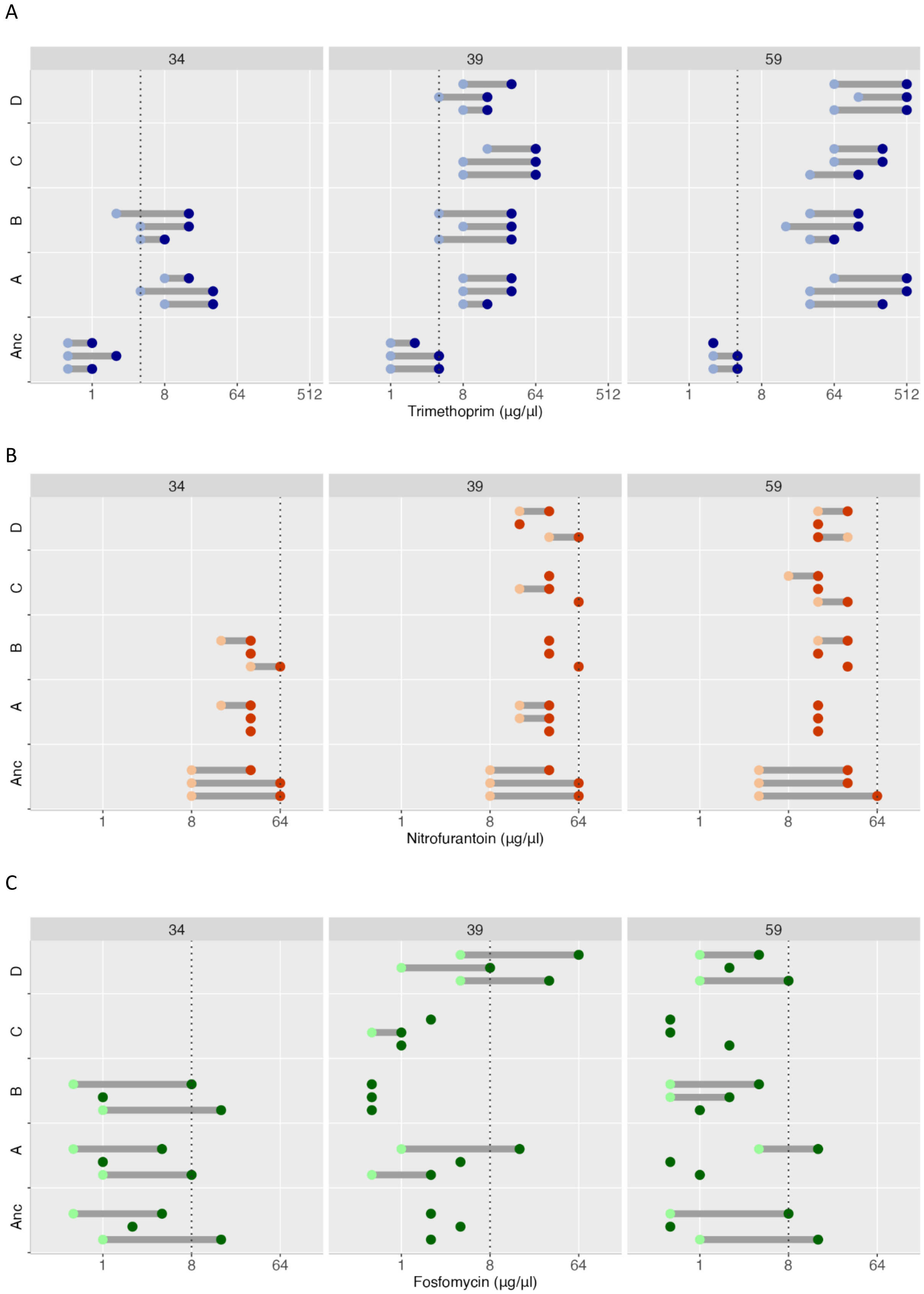

Supplementary Figure S4: Mutant selection windows of ancestor clinical isolates (Anc) and trimethoprim-resistant derivatives (A-D) for A) trimethoprim, B) nitrofurantoin, and C) fosfomycin.

Dotted lines represent the clinical breakpoint for the corresponding antibiotic. Lighter dots represent the minimum inhibitory concentration (MIC) and the darker dots represent the mutant prevention concentration (MPC). Three biological replicates were performed for each antibiotic-isolate combination (n=3).

A

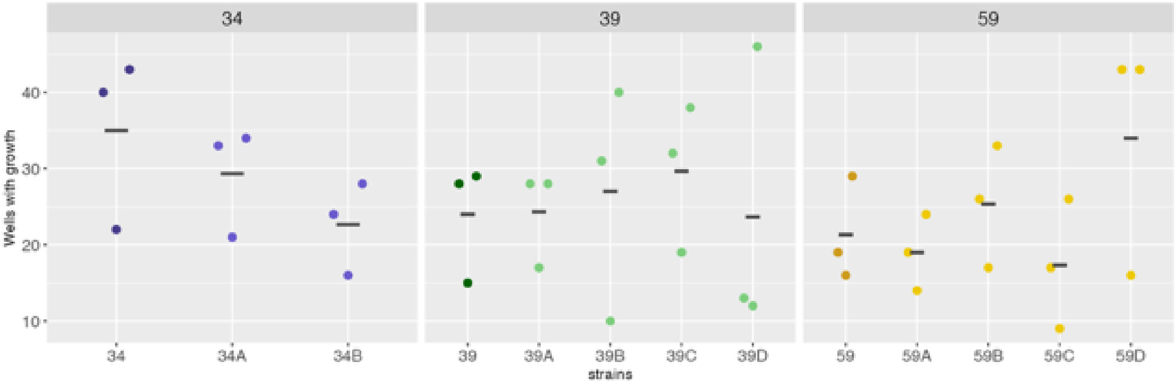

B

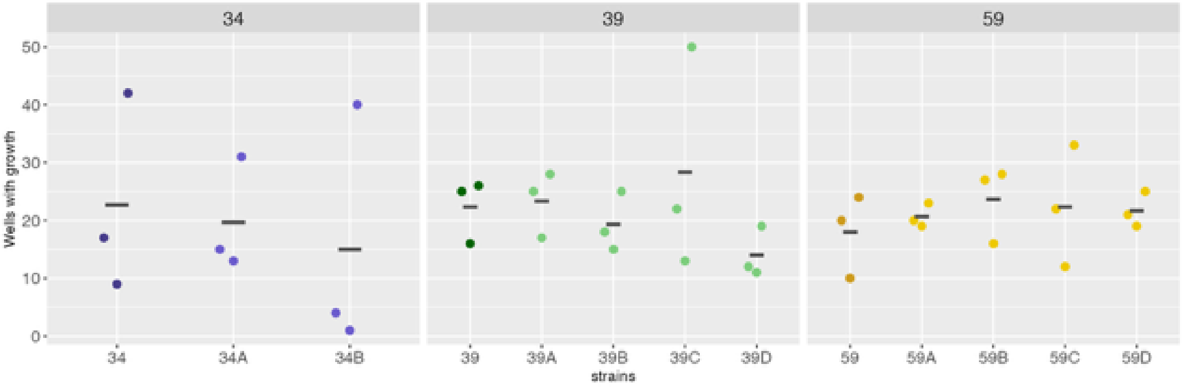

Supplementary Figure S5: Population establishment of the trimethoprim-resistance derivatives and the ancestor clinical isolates in the presence of A) nitrofurantoin and B) fosfomycin. Three biological replicates were performed for each antibiotic-isolate combination (n=3).
